# Relationship Between Problematic Smartphone Use and Graduate Students’ Research Self-Efficacy: A Moderated Mediation Model

**DOI:** 10.3390/bs14121191

**Published:** 2024-12-13

**Authors:** Peng Li, Jiangyuan Chen, Zhitong Duan, Wei Xu, Yangcun Feng

**Affiliations:** 1 Faculty of Vocational and Technical Education, Tongji University, Shanghai 201804, China; leeboom@tongji.edu.cn (P.L.);; 2 College of Foreign Languages, Shandong Normal University, Jinan 250014, China

**Keywords:** mobile phone addition, research self-efficacy, mental stress, stress mindset

## Abstract

As Generation Z youth grow up with the Internet, problematic smartphone use is growing more prevalent. This study administered questionnaires containing measures such as the Mobile Phone Addiction Index, the Research Self-Efficacy Scale, the Depression Anxiety Stress Scale, and the Stress Mindset Measure. The survey targeted 2278 graduate students and explored the mechanism through which problematic smartphone use affects research self-efficacy (RSE). The results reveal that problematic smartphone use has significant negative effects on self-efficacy, with mental stress playing a mediating role in this process; that is, problematic smartphone use lowers RSE by increasing mental stress. Meanwhile, the aforementioned negative impacts caused by problematic smartphone use are moderated by stress mindsets: the “stress-is-enhancing” mindset reduces the negative effects of smartphone use on RSE, whereas the “stress-is-debilitating” mindset amplifies these negative effects by enhancing the mediating effect of mental stress.

## 1. Introduction

With the advent of the digital age, mobile phones have gradually become an indispensable tool for shopping, entertainment, office work, finance management, and socialization. According to a previous survey, as of December 2023, the number of Internet users in China hit 1.092 billion, the Internet penetration rate reached 77.5%, the proportion of users using mobile phones to access the Internet was up to 99.9%, and the weekly Internet time rose to 26.1 h per capita [1]. The remarkable convenience offered by mobile phones, however, has negative effects. For instance, extreme emotional changes and even severe physiological reactions may arise, contributing to problematic smartphone use [2]. As the youth of Generation Z have grown up with mobile Internet, graduate students frequently use mobile phones for communication, schoolwork research, and entertainment [3]. Facing the pressure of academic research and societal expectations, they usually tend to rely on their phones for a brief escape from reality to alleviate stress. However, the long-term excessive use of mobile phones not only fails to effectively address real-life stress but also makes situations worse, as it can exacerbate mental pressure and create a vicious circle [4]. Graduate students are increasingly affected by problematic smartphone use, which translates into insufficient research output and slow development of academic skills.

Considering the seriousness of problematic smartphone use and the importance of research self-efficacy (RSE) for graduate students’ academic development, this study aimed to ascertain the relationship between problematic smartphone use and graduate students’ research self-efficacy through a moderated mediation model. By clarifying the relationship between these two aspects, we want to provide theoretical guidance for improving graduate students’ RSE.

### 1.1. Self-Efficiency and Research Self-Efficacy

Self-efficacy is defined as people’s beliefs about their capabilities to produce the desired levels of performance by exercising influence over events that affect their lives [5]. Research self-efficacy (RSE) is a more specific construct that refers to the confidence that graduate students have about their ability to successfully perform tasks related to research, such as designing studies, analyzing data, and writing research papers [6,7]. General self-efficacy can serve as a foundation for developing research self-efficacy in the academic and research context. Thus, research self-efficacy has a direct impact on graduate students’ research attitudes and behaviors, with higher self-efficacy contributing to higher research output [8], whereas low self-efficacy potentially acts as a barrier [9]. Contemporary society has witnessed the popularization of mobile digital terminals (such as smartphones), and problematic smartphone use is becoming increasingly prominent among graduate students, affecting their research self-efficacy and output. Graduate students may become deficient in face-to-face communication abilities and other social skills if they are addicted to mobile phones [10], and the consequences may include negative impacts on teamwork, academic exchanges, and collaborative publications in research activities. In addition, the prolonged use of mobile phones may harm their physical and mental health [11], leading to anxiety and sleep disorders, among other issues [12]. In the long term, their motivation to study and research can decrease, as can their RSE.

### 1.2. Review of the Relationship Between Problematic Smartphone Use and Research Self-Efficacy

Self-efficacy is a crucial element of social cognitive theory and forms the social basis for thoughts and actions. The renowned psychologist Bandura defines it as “the belief in one’s capabilities to organize and execute the courses of action required to manage prospective situations” [13]. Such belief relies on the psychosocial interactions between four constructs: performance outcomes, vicarious experiences, verbal persuasion, and physiological feedback [14]. RSE, which is derived from self-efficacy, is one of the main factors that affect students’ success in conducting research [15]. RSE denotes the confidence of an individual in utilizing acquired skills and abilities to accomplish a specific task during research activities [6]. Problematic smartphone use occupies a large part of students’ time, which means less time can be devoted to research activities. Therefore, it directly affects the development of research self-efficacy. Smartphone multitasking that is unrelated to research may hinder the cognitive processes required for learning. Researchers respond to smartphone notifications by switching tasks if they get distracted by them at work. This interrupts their research activities and diverts their mental resources to non-academic tasks [16], thus crowding out research time.

### 1.3. Review of Mental Stress and Its Mediating Effects

Research into the diverse mental, physical, and social effects of smartphone use has significantly expanded over the past decade. Among the key variables examined in the literature are the levels of stress associated with smartphone use, with evaluations of the effects of problematic versus non-problematic smartphone use [17]. Problematic smartphone use refers to over-reliance on mobile phones to the extent that it interferes with daily life and work routines. Previous studies have focused on mental stress as an antecedent variable for problematic smartphone use [18,19,20]. However, for “Generation Z” students, as they have been exposed to digital technology since childhood, their dependence on technology is an important part of their growth; therefore, most of them are dependent on mobile phones and may develop problematic smartphone use.

Graduate students are under multiple external stressors, including from school, work, and family [21]. To cope with these stressors, they generally seek psychological solace and escape through mobile phone use [22]. However, the excessive, long-term use of mobile phones not only fails to effectively relieve actual stress, but it can also exacerbate mental stress and create a vicious circle. Rosen’s team observed that such addiction affects psychological states and causes agitation, anxiety, depression [23], and a severe lack of self-confidence. Meanwhile, Lepp, Barkley, and Karpinski noted that higher stress and anxiety and lower academic performance are associated with higher mobile phone use [24]. Deteriorating mental health is a consequence of problematic smartphone use. Sustained mental stress depletes graduate students’ limited mental resources [25], reduces their attention and commitment to research activities, and ultimately affects their research self-efficacy. Moreover, their research self-efficacy is further reduced with greater dependence on mobile phones, leading to higher mental stress.

### 1.4. Review of Stress Mindset and Its Moderating Effects

Stress mindset refers to an individual’s mental attitude and behavioral tendency when facing stress, challenges, or difficulties and can be categorized as a stress-is-enhancing mindset or a stress-is-debilitating mindset [26]. Individuals with the former mindset are optimistic, self-confident, and adaptable. They are more determined and proactive in seeking solutions to problems when facing difficulties; this fosters their adaptation and growth. In contrast, individuals with a stress-is-debilitating mindset tend to be negative, anxious, and avoidant. They may experience feelings of helplessness, frustration, and anxiety, which hinder their ability to effectively solve problems. Therefore, evasion and avoidance are their responses to hardships, resulting in worsening crises and mental health problems. Relatively speaking, adolescents with a stress-is-debilitating mindset suffer significantly higher levels of distress under adversity [27].

Problematic smartphone use manifests externally as persistent, immersive, and long-duration use, often resembling a compulsion or an addiction [17]. This theory of problematic smartphone use is influenced by theoretical models of internet addiction, and the mechanisms are thought to involve the route through which smartphone use can cause psychopathological symptoms such as stress, anxiety, and depression [28]. Therefore, problematic smartphone use creates an imbalance in implicitly internal mental states, resulting in pronounced damage to the mental and social functioning of individuals [29]. Those who possess a stress-is-enhancing mindset are capable of rationally dealing with the positive effects of excessive mobile phone use (e.g., the diversionary effects of entertainment and socialization), thereby reducing mental stress. On the contrary, a stress-is-debilitating mindset forces individuals to regard excessive mobile phone use as a waste of time, and this would spawn an increase in anxiety, guilt, and mental stress.

Regarding RSE, stress mindsets influence how individuals appraise research-related challenges. Those with a stress-is-enhancing mindset may view research obstacles as opportunities for growth, thereby bolstering their confidence in handling research tasks [30]. A positive stress mindset can enhance motivation and persistence in research activities, as individuals are more likely to engage with challenging tasks and persist in the face of difficulties [31]. By framing stress as a challenge rather than a threat, individuals can enhance their self-efficacy beliefs, which is associated with greater engagement with and success in research endeavors. Regarding the effects of problematic smartphone use on research self-efficacy, individuals who possess a stress-is-enhancing mindset enjoy more of the resource advantages and peer support offered by mobile phone use, leading to better research self-efficacy. In contrast, those with a stress-is-debilitating mindset are less likely to reap benefits from using mobile phones, and their research efficacy will be further weakened by the uncertainty of research outputs and the long cycle of publication.

## 2. Goal of the Study

**Figure 1:**
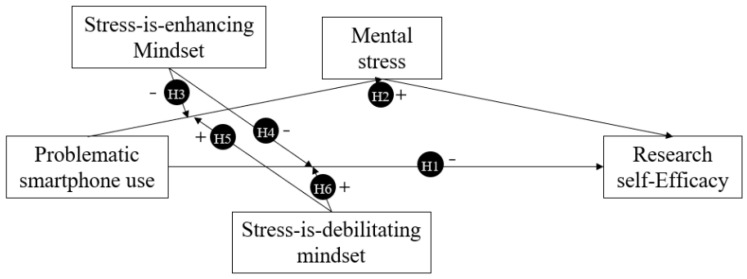
Hypothetical model.

This study aimed to measure the mechanisms of problematic smartphone use on research self-efficacy among graduate students and to further examine the mediating effects of mental stress and the moderating effects of stress mindsets. Figure 1 shows the moderated mediation model for the study. The following hypotheses were tested in this study:

**H1.**  *Mobile phone addictive behaviors decrease students’ research self-efficacy.*

**H2.**  *Mental stress can partially mediate the effects of problematic smartphone use on research self-efficacy.*

**H3.**  *A stress-is-enhancing mindset can reduce the effects of problematic smartphone use on mental stress.*

**H4.**  *A stress-is-enhancing mindset can mitigate the effects of problematic smartphone use on research self-efficacy.*

**H5.**  *A stress-is-debilitating mindset can enhance the effects of problematic smartphone use on mental stress.*

**H6.**  *A stress-is-debilitating mindset can increase the effects of problematic smartphone use on research self-efficacy.*

## 3. Methods

### 3.1. Participants and Setting

Employing a survey questionnaire method, a proportionate stratified random sampling procedure was used to collect data from students in Chinese universities at different levels. The questionnaires were distributed online nationwide through the Wenjuanxing App (version 2.2.6) to various universities in China to determine the mental stress, research self-efficacy, problematic smartphone use, and stress mindsets of graduate students. After being told that their personal information would be strictly protected and they could withdraw without any consequences, each participant was invited to complete an online survey questionnaire wherein the anonymity of each response was guaranteed. Finally, a total of 2278 valid questionnaires were collected and organized after applying the detailed guidelines of the study. The participants were 1464 full-time and 814 part-time graduate students. The gender balance was fair, with 1114 (48.90%) male participants and 1164 (51.10%) female participants. In addition, 372 (16.33%), 491 (21.55%), and 472 (20.72%) participants were, respectively, first-, second-, and third-year master’s degree students. Furthermore, the numbers of first-, second-, third-, fourth-, and fifth-year students in doctoral programs were 265 (11.63%), 216 (9.48%), 255 (11.19%), 120 (5.27%), and 87 (3.82%), respectively.

### 3.2. Measures

The research variables in this study were mental stress, research self-efficacy, problematic smartphone use, and stress mindsets. To scientifically and effectively measure the research variables, the following scales were organized and compiled in Chinese for the questionnaire based on previous research efforts.

#### 3.2.1. Mental Stress Scale

The items for this scale were obtained from the questions in the stress section of the Depression Anxiety Stress Scale (DASS) [32]. We selected seven items and adopted a five-point scoring method, where one represents “a complete lack of compliance” and five denotes “complete compliance”. The scale comprises five dimensions: difficulty relaxing, nervous excitement, distraction, allergic reaction, and impatience. The Cronbach’s alpha was 0.902 for this scale, indicating high reliability and dependability.

#### 3.2.2. Research Self-Efficacy Scale

For this scale, seven questions from Black’s revised Research Self-Efficacy Scale (RSES) [33] were selected, and the same scoring method as that for the Mental Stress Scale was adopted. Individuals’ scores on this scale were proportional to their research self-efficacy. This scale can be divided into three dimensions, namely, research methodology and dissemination, regulations and organizations, and interpersonal aspects. The reliability for this scale was considerably high, with a Cronbach’s alpha of 0.914.

#### 3.2.3. Problematic Smartphone Use Scale

We measured behaviors associated with problematic smartphone use using 12 questions adopted from Leung’s revised Mobile Phone Addiction Index (MPAI) [34]. Similar to the abovementioned scoring system, one denotes “almost never” and five denotes “always”. The higher an individual’s score on this scale, the more dependent they are on their mobile phone. Four dimensions form this scale: inefficacy, avoidance, loss of control, and withdrawal. The Cronbach’s alpha was 0.959, showing high reliability.

#### 3.2.4. Stress Mindset Measure

The present study selected six questions from the Stress Mindset Measure (SMM) [26] developed by Crum et al. to measure stress mindsets. We used the same scoring method, where one means “strongly disagree” and five denotes “strongly agree”. The two-side scale assesses the stress-is-enhancing mindset and stress-is-debilitating mindset. The reliability was not as remarkable for this scale as for others, but it remained relatively good, with a Cronbach’s alpha of 0.810.

#### 3.2.5. Statistical Analysis

This study used SPSS 27.0 software for data analysis, and Harman’s single-factor test was used to check for common method biases. Based on the theory of testing mediating effects proposed by Wen Zhonglin et al., this study analyzed the chain of mediating effects and moderating effects using the built-in Process macro of SPSS [35].

## 4. Results

### 4.1. Test of Common Method Biases

Self-report data collection methods are often subject to common method biases, so this study employed anonymization and reverse scoring of some entries during the procedural aspects. In the next step, Harman’s single-factor test was applied to check for common method biases after data recovery. The results revealed four factors with eigenvalues greater than 1, and the explained variance of the first factor accounted for 42.425% (less than 50%) [36]. In summary, no significant problems related to common method biases were identified.

### 4.2. Mean, Standard Deviation, and Correlation Matrix for the Study Variables

The descriptive statistics and correlation analysis results revealed the following (see Table 1): problematic smartphone use was highly and negatively correlated with research self-efficacy, stress-is-enhancing mindset, and stress-is-debilitating mindset. Mental stress had a negative relationship with research self-efficacy and the two mindsets, but it had a positive correlation with problematic smartphone use. Research self-efficacy was highly negatively correlated with problematic smartphone use and the stress-is-debilitating mindset, whereas it was positively correlated with the stress-is-enhancing mindset.

### 4.3. Test of Hypothetical Model

#### 4.3.1. Mediation Model Test

The PROCESS 4.1 plug-in for SPSS 27.0 was used to test the mediating effect [37] according to the bootstrap method proposed by Hayes: model 4 with a sample size of 5000 at the 95% confidence interval. Problematic smartphone use, research self-efficacy (TPI at 5000 ms), and mental stress were, respectively, set as the independent variable X, dependent variable Y, and mediating variable M after controlling for gender, grade, study mode, and degree type. The bootstrap results reveal (see Table 2 and Table 3) that the indirect effect of the mediating variable does not contain 0 (effect size = −0.079, standard error = 0.014, 95% confidence interval = [−0.107, −0.052]). Additionally, after including the mediating variable of mental stress in the model, the direct effect of problematic smartphone use on research self-efficacy remains notable, as 0 is excluded from its 95% confidence interval (effect size = 0.435, standard error = 0.019, 95% confidence interval = [−0.471, −0.398]). Therefore, in light of Zhao et al.’s theory [38], these results confirm the partial mediating effect of mental stress on the relationship between problematic smartphone use and research self-efficacy, thus supporting H1 and H2.

#### 4.3.2. Moderated Mediation Model Test

This study hypothesized that stress mindsets modulate the pathway through which problematic smartphone use affects research self-efficacy, which is mediated by mental stress. Model 10 in PROCESS V4.1 was used to test the moderating effects of stress mindset, while controlling for gender, grade, study mode, and degree type. The product term of problematic smartphone use and stress-is-enhancing mindset is a nonsignificant predictor of mental stress but a significant predictor of research self-efficacy. The product term of problematic smartphone use and stress-is-debilitating mindset is a weak predictor of research self-efficacy but a strong predictor of mental stress. This implies that a stress-is-enhancing mindset plays a moderating role when problematic smartphone use affects research self-efficacy, and a stress-is-debilitating mindset can have a similar effect when problematic smartphone use impacts mental stress (see Table 4).

**Figure 2:**
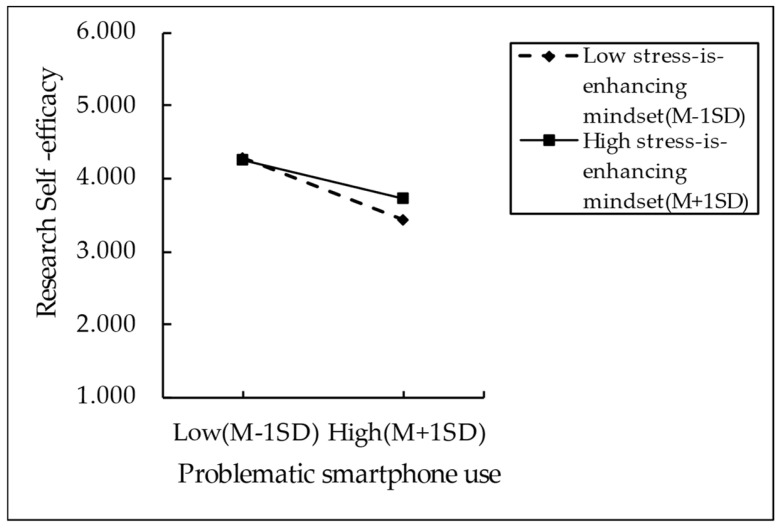
Moderating effects of stress-is-enhancing mindset.

**Figure 3:**
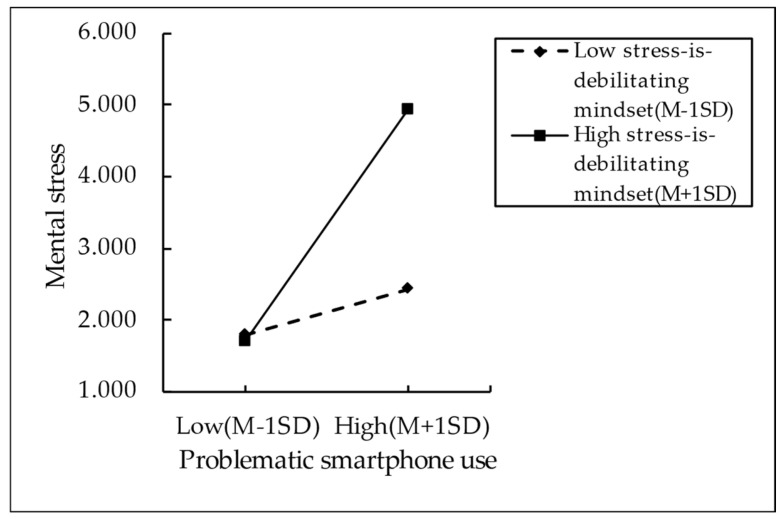
Moderating effects of stress-is-debilitating mindset.

To further elucidate the moderating effects of stress mindsets, we categorized participants into high-scored (M + 1SD) and low-scored (M − 1SD) groups based on their scores on the Stress Mindset Measure (encompassing both stress-is-enhancing mindset and stress-is-debilitating mindset). Accordingly, simple slope analyses were conducted, bounded by one standard deviation below and above the mean (see Figure 2 and Figure 3 for specific results). Figure 2 reveals that the negative predictive effects of problematic smartphone use on research self-efficacy are significant in the group with a lower level of stress-is-enhancing mindset (M − 1SD), with a slope value of −0.439, a t-value of −16.265, and a *p*-value of less than 0.001. In contrast, in the group with a higher level of such a mindset (M + 1SD), the negative effects are relatively weak, with a slope value of −0.279, a t-value of −9.407, and a *p*-value of less than 0.001. This provides evidence that the negative impacts of problematic smartphone use on an individual’s research self-efficacy tend to diminish with a higher level of the stress-is-enhancing mindset. Figure 3 reveals a significant and positive predictive relationship between problematic smartphone use and mental stress in the group with a high level of stress-is-debilitating mindset (M + 1SD), with a slope value of 0.484, a t-value of 18.072, and a *p*-value of less than 0.001. In contrast, in the group of participants with a lower level of stress-is-debilitating mindset (M − 1SD), problematic smartphone use is only a weak positive predictor of mental stress, with a slope value of 0.332, a t-value of 12.174, and a *p*-value of less than 0.001. This suggests that the positive predictive effects of problematic smartphone use on mental stress tend to increase in individuals with a high level of stress-is-debilitating mindset. In addition, as presented in Table 5, the mediating effect of mental stress exhibits a downward trend at different levels of stress-is-debilitating mindset. In other words, the “stress-is-debilitating” mindset amplifies the negative effects of smartphone addiction on research self-efficacy by enhancing the mediating effect of mental stress.

**Figure 4:**
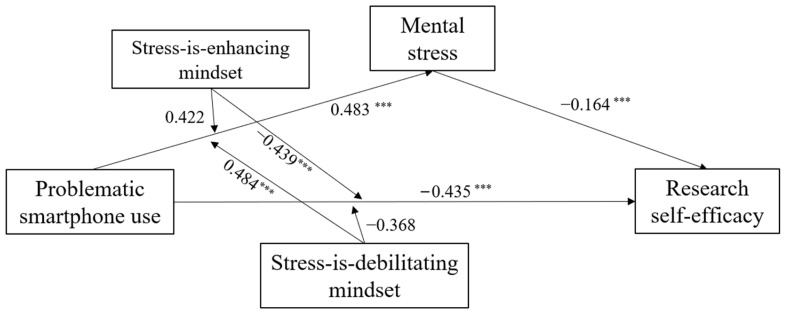
Resulting model. Note: *** *p* < 0.001.

The modified structural equation model of this study is shown in Figure 4.

## 5. Discussion

This study constructed a moderated mediation model, where mental stress was the mediating variable and stress mindset was the moderating variable. It has been observed that excessive engagement with digital devices may detract from mental focus and resilience, and problematic smartphone use appears to correlate strongly with reduced research self-efficacy. Specifically, problematic smartphone use influences graduate students’ research self-efficacy via mental stress as the mediating variable. Finally, the negative impacts of smartphone addiction on research self-efficacy can be lowered by interfering with this process through a positive stress mindset.

### 5.1. Problematic Smartphone Use Has Significant Negative Impacts on Research Self-Efficacy

The results suggest that the direct effects of problematic smartphone use on research self-efficacy have a value of −0.435. This validates H1: problematic smartphone use has significant negative effects on graduate students’ research self-efficacy. This finding is largely consistent with common sense, and the causes can be attributed to several factors. First, smartphones are often seen as a source of distraction that may affect task performance [39]. Additionally, research evidence indicates that smartphones can replace deep thinking and foster cognitive miserliness [40]. Thus, graduate students may have trouble concentrating in class due to this distraction. Additionally, dependence on mobile devices means that graduate students develop a habit of multitasking, making it difficult to sustain research work that requires high concentration [23]. Second, the use of mobile phones is an “invisible” drain on time. Smartphone addiction can lead individuals to spend substantial amounts of time on mobile phones unconsciously [41], thus reducing the energy devoted to research activities, which directly affects the development of research self-efficacy. In addition, a longer time spent on smartphones also has negative impacts on a range of offline activities, varying from relationships to academic performance [42]. Third, the “fear of missing out” (FOMO) mentality is triggered by problematic smartphone use. Generation Z has grown up with the Internet and digital devices and is reluctant to miss out on what is happening online; they have strong desires to maintain interactions with the rest of the world. Therefore, they are likely to suffer from inattention and make insufficient preparations for scientific research [43]. Fourth, the fragmented information that students can obtain through mobile phones does not offer deep learning experiences and a sense of gain. Students may get bored when they lack motivation, and smartphone apps could provide a quick and tantalizing escape [44]. Immersed in the age of fragmentation, the excessive use of mobile phones exerts a dulling effect on students, and this over-reliance deteriorates people’s ability to write, remember, and deeply think [45]. These factors affect the progress or even the results of research and, ultimately, the development of research self-efficacy.

### 5.2. Mental Stress Mediates the Relationship Between Problematic Smartphone Use and Research Self-Efficacy

The results of this study reveal the partial mediating effect of mental stress, with an effect value of −0.107, which validates H2: mental stress has a mediating effect on the impact of problematic smartphone use on graduate students’ research self-efficacy. Previous studies have revealed that problematic smartphone use is a positive predictor of mental stress [46]. Graduate students are often under tremendous research pressure, and when they return to the real world after engaging their minds in the virtual space, they tend to feel regret and blame themselves for wasting time and letting their research tasks become more urgent. As mental pressures [47] and doubt regarding their research abilities increase, their research self-efficacy declines. Additionally, the excessive use of mobile phones may be emotionally damaging [48], leading to anxiety and depression. These issues affect an individual’s mental state, interfere with their thought processes, and influence their decision-making and problem-solving abilities [49], thereby weakening their self-confidence in accomplishing research tasks and undermining their motivation and initiative when facing research challenges [50]. In addition, each individual has limited mental resources, which will be depleted with sustained stress [25]. Such depletion not only exhausts and stresses students, it may also weaken their focus and commitment to research tasks [51]. There is substantial research suggesting that problematic smartphone use can impair executive functions such as attention, working memory, and cognitive control. These cognitive impairments could also play a critical role in lowering research self-efficacy, as they may affect a student’s ability to concentrate, organize tasks, and engage in deep thinking, which are essential for research productivity [52]. As a high degree of concentration and sustained cognitive effort is required in research, the persistent effects of mental stress may reduce the amount and quality of time that students devote to their research activities. As a consequence, their expectations of the quality and efficiency in finishing research tasks and, ultimately, their research self-efficacy will all be negatively affected.

### 5.3. Stress Mindsets Present Different Moderating Effects

#### 5.3.1. Effects of Stress-Is-Enhancing Mindset

The results suggest that a stress-is-enhancing mindset could modulate the effects of problematic smartphone use on research self-efficacy (path: problematic smartphone use → research self-efficacy), with an effect value of −0.439. However, it does not have a significant effect on the relationship between problematic smartphone use and mental stress (path: problematic smartphone use → mental stress). Therefore, H4 is supported, whereas H3 is not. For the path illustrating the direct impact of “problematic smartphone use → research self-efficacy,” the negative effects of problematic smartphone use on research self-efficacy are weakened by a stress-is-enhancing mindset. This finding is largely consistent with previous research on the stress-is-enhancing mindset, suggesting that people can develop a positive mindset that is conducive to better mental health in the face of stress or challenges. This mindset can help people cope better with life difficulties and enhance their adaptability and resilience [26]. Individuals with a stress-is-enhancing mindset can reap more resource advantages and peer support, among other benefits, associated with mobile phone use. Therefore, the negative impacts of problematic smartphone use on research self-efficacy may be lower. However, an analysis of the data collected for this study suggests that the stress-is-enhancing mindset cannot modulate the effects of problematic smartphone use on mental stress. Specifically, this mindset fails to alleviate the problem of worsening mental stress due to problematic smartphone use, which may be because while the stress-is-enhancing mindset encourages individuals to face challenges more actively, it may also make them more sensitive to stress. Even minor stress from problematic smartphone use might be noticed and taken seriously by those with the stress-is-enhancing mindset, which is associated with a slight, although non-significant, increase in mental stress.

#### 5.3.2. Effects of Stress-Is-Debilitating Mindset

The results of this study indicate that the stress-is-debilitating mindset could modulate the effects of problematic smartphone use on mental stress, with an effect value of 0.484. However, the moderating effects of problematic smartphone use on research self-efficacy are not significant, supporting H5 but not H6. For the path of “problematic smartphone use → mental stress”, problematic smartphone use has significant positive effects on mental stress, which can be strengthened by the stress-is-debilitating mindset. Such a mindset may worsen the disturbance of negative feelings based on the emotional and physiological states experienced in a given environment [53]. The stress-is-debilitating mindset enhances the negative effects of problematic smartphone use on mental stress. Its moderating effects occur largely because excessive use of mobile phones may contribute to emotional problems, such as anxiety and depression. These problems affect an individual’s psychological state and worsen mental stress. However, the lack of a significant moderating effect of the stress-is-debilitating mindset in the pathway of “problematic smartphone use → research self-efficacy” suggests that this type of mindset does not exacerbate the decline in research self-efficacy due to problematic smartphone use. These findings seem contradictory but might be explained by the “mandatory” nature of scientific research. For all graduate students, research is closely tied to graduation, and the demands of academic realities must be first considered even if problematic smartphone use is severe. Even with a stress-is-debilitating mindset, graduate students might be aware of the negative impact of problematic smartphone use on their research activities, and motivated by self-interest, they might try to control their phone use to mitigate its damage to their research efficiency. However, with a lack of effective coping strategies and psychological resilience, this protective effect might not be significant.

#### 5.3.3. Further Analysis of Stress Mindsets’ Moderating Effects

The main results of this study can be summarized as follows: which stress mindset (stress-is-debilitating or stress-is-enhancing) has a significant impact is determined by the nature of the variable affected by problematic smartphone use. If the variable is negative (such as mental stress), then a negative stress mindset (stress-is-debilitating) will play a moderating role, and if the variable is positive (such as RSE), then a positive stress mindset (stress-is-enhancing) will play a moderating role. These findings indicate that the role of stress mindsets varies in different contexts. In the case of problematic smartphone use increasing mental stress, the stress-is-debilitating mindset significantly worsens the issue, while in the case of problematic smartphone use reducing research efficiency, the stress-is-enhancing mindset significantly alleviates the problem. This highlights the importance of adjusting stress mindset when dealing with problematic smartphone use and its impacts.

## 6. Limitation and Future Research

The study focused exclusively on graduate students, which limits the generalizability of the findings to other populations, such as undergraduate students or working professionals. The specific academic environment and pressures faced by graduate students might not reflect the experiences of other groups. In addition, the findings may be influenced by cultural factors specific to the region or country where the study was conducted. Cultural attitudes towards mobile phone use and stress may vary, affecting the applicability of the findings to different cultural settings.

Another very important limitation of this study is its use of a cross-sectional design. This design collects data at a single point in time, making it impossible to determine causal relationships between variables. The problematic smartphone use can be both an independent variable and a dependent variable. It can serve as a solution to psychological issues, but it can also act as a triggering factor. It is entirely possible that lower research self-efficacy leads to increased mental stress, which might, in turn, result in problematic smartphone use as a coping mechanism. Some studies indicated the component of urgency to be a robust predictor of problematic mobile phone use [54]. Some forms of psychopathology, including stress and depression, have also been found in longitudinal studies as outcomes of problematic smartphone use [19,55]. Additionally, smartphones may serve as instruments that guarantee personal safety, especially for individuals with panic disorders [56]. In the absence of interruption of such a maladaptive mechanism, as addressed by Kim, Seo, and David [57], there may be a risk for a vicious cycle between psychopathology and smartphone addiction, such that an increased level of perceived distress may lead to increased smartphone use which, in turn, may inadvertently increase the level of stress. The phenomenon known as technostress [58] concerns the negative outcomes and affective consequences derived from an overload of information and communication made available by computer technologies. Innovative research carried out in Korea by Lee, Chang, Lin, and Cheng [59] confirmed that compulsive and continuous smartphone use was positively associated with users’ technostress.

Future research could explore interventions aimed at reducing problematic smartphone use and its negative effects on research self-efficacy. This could include stress management programs or digital detox initiatives. As technology evolves, future studies should consider the impact of new digital platforms and applications on problematic smartphone use and related psychological outcomes.

## Figures and Tables

**Table 1:** Descriptive statistics and related analysis.

	Mean	Standard Deviation	Problematic Smartphone Use	Mental Stress	Research Self-Efficacy	Stress-Is-Enhancing Mindset	Stress-Is-Debilitating Mindset
Problematic smartphone use	2.413	0.963	1	0.549 **	−0.564 **	−0.497 **	−0.409 **
Mental stress	2.190	0.857	0.549 **	1	−0.430 **	−0.376 **	−0.320 **
Research self-efficacy	3.883	0.889	−0.564 **	−0.430 **	1	0.419 **	−0.350 **
Stress-is-enhancing mindset	3.864	0.887	−0.497 **	−0.376 **	0.419 **	1	−0.675 **
Stress-is-debilitating mindset	2.494	0.886	−0.409 **	−0.320 **	−0.350 **	−0.675 **	1

Note: ** correlation is significant at the 0.01 level (2-tailed).

**Table 2:** Test of the mediating effects of mental stress.

		X	M	R^2^	F
Model 1	β	0.483		0.311	171
	t	31.052			
	LLCI	0.453			
	ULCI	0.514			
Model 2	β	−0.435	−0.164	0.352	175
	t	−23.235	−7.742		
	LLCI	−0.471	−0.205		
	ULCI	−0.398	−0.122		
Model 3	β	−0.514		0.335	190
	t	−32.359			
	LLCI	−0.545			
	ULCI	−0.482			

Note: X—problematic smartphone use; Y—research self-efficacy; M—mental stress.; Model 1: X predicts M; Model 2: X predicts Y; Model 3: X and M predict Y; LLCI—Lower Limit Confidence Interval; ULCI—Upper Level of Confidence Interval.

**Table 3:** Breakdown of total, direct, and mediating effects.

	Effect Value	Standard Deviation	LLCI	ULCI	Ratio in Total Effect
Total effect	−0.514	0.016	−0.545	−0.482	
Direct effect	−0.435	0.019	−0.471	−0.398	
Mediating effect	−0.079	0.014	−0.107	−0.052	0.154

**Table 4:** Test of the moderating effects of stress mindsets.

Outcome	Predictor	*β*	LLCI	ULCI	*t*
Mental stress	Problematic smartphone use	0.408	0.373	0.443	22.664
	Stress-is-enhancing mindset	−0.048	0.052	−0.097	−1.944
	Problematic smartphone use × stress-is-enhancing mindset	−0.016	0.478	−0.06	−0.709
	Stress-is-debilitating mindset	0.04	0.09	−0.006	1.694
	Problematic smartphone use × stress-is-debilitating mindset	0.088	0.042	0.133	3.776
	R^2^	0.335			
	F	114.354			
Research self-efficacy	Problematic smartphone use	−0.359	−0.398	−0.320	−17.971
	mental stress	−0.127	−0.168	−0.086	−6.032
	Stress-is-enhancing mindset	−0.078	0.029	0.127	3.120
	Problematic smartphone use × stress-is-enhancing mindset	0.09	0.046	0.135	3.973
	Stress-is-debilitating mindset	−0.037	−0.083	0.009	−1.598
	Problematic smartphone use × stress-is-debilitating mindset	−0.010	−0.056	0.036	−0.427
	R^2^	0.380			
	F	125.992			

**Table 5:** Direct and mediating effects at different levels of stress mindsets.

	Stress Mindset	Effect Value	Standard Deviation	LLCI	ULCI
Direct effect (modulation by stress-is-enhancing mindset)	−0.887 (M − 1SD)	−0.439	0.027	−0.492	−0.386
	0 (M)	−0.359	0.020	0.398	−0.320
	0.887 (M + 1SD)	−0.279	0.030	−0.337	−0.221
Mediating effect of mental stress (modulation by stress-is-debilitating mindset)	−0.887 (M − 1SD)	−0.042	0.010	−0.064	−0.023
	0 (M)	−0.052	0.012	−0.076	−0.029
	0.887 (M + 1SD)	−0.061	0.015	−0.091	−0.034

## Data Availability

The data presented in this study are available from the corresponding author upon request.
